# Tolerance Considerations for MHMIC Manufacturing Process at Millimeter-Wave Band

**DOI:** 10.3390/s24082486

**Published:** 2024-04-12

**Authors:** Chaouki Hannachi, Matthieu Egels, Philippe Pannier, Serioja Ovidiu Tatu

**Affiliations:** 1 Institut Matériaux Microélectronique Nanosciences de Provence (IM2NP), CNRS, UMR 7334, Aix-Marseille Université, 13000 Marseille, France; matthieu.egels@im2np.fr (M.E.); philippe.pannier@im2np.fr (P.P.); 2 Institut National de la Recherche Scientifique (INRS), Centre Énergie Matériaux Télécommunications, Montréal, QC H2X 1E3, Canada

**Keywords:** analysis, accuracy, bonding, calibration, error, manufacturing, millimeter-wave, MHMICs, resistors, radial stub, thin-layer, tolerances, TRL, transmission lines, uncertainties

## Abstract

This paper investigates the manufacturing uncertainties at a 60 GHz millimeter-wave band for the monolithic hybrid microwave integrated circuits (MHMIC) fabrication process. It specifically deals with the implementation tolerances of thin-film gold microstrip transmission lines, titanium oxide thin-layer resistors, microstrip quarter-wavelength radial stubs, and active device implementation using the gold-bonding ribbons. The impacts of these manufacturing tolerances are assessed and experimentally quantified through prototyped MHMIC circuits. This allows us, on one hand, to identify the acceptable amount of dimensional variation enabling reasonable performances. On the other hand, it aims to establish a relationship between the manufacturing tolerances and the circuit parameters to provide more flexibility for the tolerance compensation and accuracy enhancement of the MHMIC fabrication processes.

## 1. Introduction

Since its introduction in 1960, the technology of Miniature Hybrid Microwave Integrated Circuits (MHMICs) has constantly advanced, and it has widely been used in many RF/microwave applications [1,2,3,4,5]. It has been particularly useful for small-scale production and prototyping purposes, thus resulting in a significant reduction in production costs and development times. MHMICs are regarded as a matured technology, integrating various transmission lines (coplanar waveguide, microstrip, slot-lines) [6,7], as well as a large number of passive components typically found in gallium arsenide monolithic microwave integrated circuits (GaAs MMICs). Those passive components include, but are not limited to, thin-film resistors, air-bridges, metal insulator metal (MIM) capacitors, and planar inductors [8,9]. They are mostly implemented on less expensive and low-loss thin-film alumina ceramic substrates [9]. However, active devices or elements are connected to the circuits separately with wire or ribbon bonds since they are not formed in or on the substrate [10,11,12,13].

Although MHMIC technology today offers well-proven performance at frequencies above 60 GHz due to its high-density integration potentials [2], it remains relatively sensitive to manufacturing tolerances. Several MHMICs have been reported in the literature in recent years, covering a wide range of frequencies and applications [1,14,15]. The vast majority of proposed works were design-oriented. and there is no specific study that has dealt with the manufacturing process tolerance and its limits. That kind of study is, in fact, required to enable the quantification of process inaccuracies, which allows MHMICs’ performance improvement. For instance, the implementation of MHMIC termination loads and isolation resistors is sensitive to fabrication tolerances, resulting in phase and amplitude imbalance, especially when it comes to multiport circuit design. Those termination loads are typically integrated using a 100 Ω per square titanium oxide thin layer, along with quarter-wavelength radial stubs to enable proper impedance matching while avoiding metallization via holes.

In this paper, the sensitivity to the tolerances of the MHMIC manufacturing process is investigated at the millimeter-wave frequency band. The tolerance analysis specifically addresses the implementation tolerances of thin-film gold microstrip transmission lines, titanium oxide thin-layer resistors, microstrip quarter-wavelength radial stubs, and active device implementation using the gold-bonding ribbons. It turns out that manufacturing tolerance optimization provides enhanced performances in terms of amplitude and phase balances. Therefore, tolerance quantification enables fabrication process improvement, as well as the establishment of a relationship between the manufacturing tolerances and the design parameters. This study aims to provide more flexibility for the tolerance compensation and accuracy enhancement of the MHMIC fabrication process. 

## 2. On-Wafer Calibration Techniques and Standards

To achieve an accurate assessment of manufacturing uncertainties in the designed MHMIC circuits as part of this study, it is necessary to select an appropriate calibration technique. This task leads to effectively correcting on-wafer measurement errors due to the non-ideal characteristics of the cables and probes at the operating millimeter-wave frequency band. The most commonly used calibration techniques for millimeter-wave on-wafer measurements are LRM (Line–Reflect–Match), LLRM (Line–Line–Reflect–Match) and TRL (Thru–Reflect–Line). LRM and LLRM calibrations require match standards, which are difficult to achieve accurately at millimeter-wave frequencies. We then chose the TRL calibration technique by implementing our calibration kit with the circuits to be characterized instead of using an expensive commercial impedance standard substrate (ISS).

To connect the Ground Signal Ground (GSG) 150 μm probes to the TRL standard ports, a microstrip line transition to a coplanar line was designed and implemented at each standard access. In the adopted approach, an RF short circuit was made by a quarter-wave sector, avoiding via holes metallization that is difficult to repeatably achieve at millimeter-wave frequencies [16].

**Figure 1:**
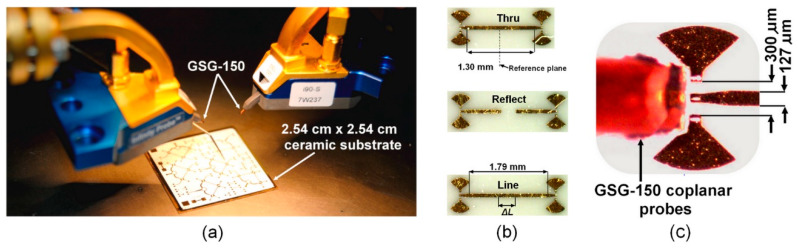
On-wafer two-port circuit characterization in (**a**), the microphotographs of the manufactured TRL calibration standards in (**b**), and the GSG 150 μm Infinity probe on the access of the microstrip line/coplanar line transition in (**c**).

The photograph of the on-wafer two-port circuit characterization, the microphotographs of the TRL calibration standards, and the GSG 150 μm probe on the access of the microstrip line/coplanar line transition are, respectively, illustrated in Figure 1a–c, shown above. The TRL calibration kit consists of a line thru (T), two open circuits (reflect R), and a line (L). Due to the fragility of the thin layer of gold (1 μm), multiple identical standards were manufactured on the same 2.54 cm by 2.54 cm alumina ceramic substrate (ε_r_ = 9.5, h = 127 μm) to ensure the repeatability and success of calibrations and measurements.

The calibration was carried out by connecting the Cascade Microtech Infinity GSG 150 μm probe to the three standards, starting with a direct connection (Thru) between the two ports that had the property of fixing the reference plane in the middle of its length. Then, we connected an open-circuit line (Reflect), and we ended with a transmission line (Line). The length of the latter limits the measurement frequency band, and it is estimated by the relationship in (1). This is relative to the “Thru” line, which is considered to have zero length. It is noteworthy that our measurement setup is designed to operate in the frequency band of 60 to 90 GHz. Under those considerations, the corresponding wavelengths are, respectively, *λ_g_*_1_ = 1.9 mm and *λ_g_*_2_ = 1.23 mm [2].

(1) $$\frac{\lambda_{g1}}{4}<∆L<\frac{\lambda_{g2}}{2}\;$$

where Δ*L* is the length difference between the “Line” and “Thru” standards. In that respect, we have selected lengths of 1.305 mm for the “Thru” line, and a length of 1.798 mm for the “Line” line. Then, Δ*L* is equal to 0.493 mm, ranging between *λ_g_*_1_/4 = 0.475 mm and *λ_g_*_2_/2 = 0.615 mm. Therefore, the “Thru” and “Line” line lengths enable it to cover the entire frequency band, from 60 to 90 GHz, without a phase ambiguity problem.

## 3. Manufacturing Tolerance Analysis of the Gold Thin-Film Microstrip Line

The loss assessment of the gold thin-film microstrip line is an important operation to optimize the manufacturing process of MHMIC circuits at millimeter-wave bands. It should be noted that most substrate manufacturers do not provide information covering the millimeter-wave range. Quantifying losses over the operating frequency band between 60 and 70 GHz is then performed by measuring the insertion losses of two gold thin-film microstrip lines with, respectively, lengths of 2.486 mm and 2.914 mm.

**Figure 2:**
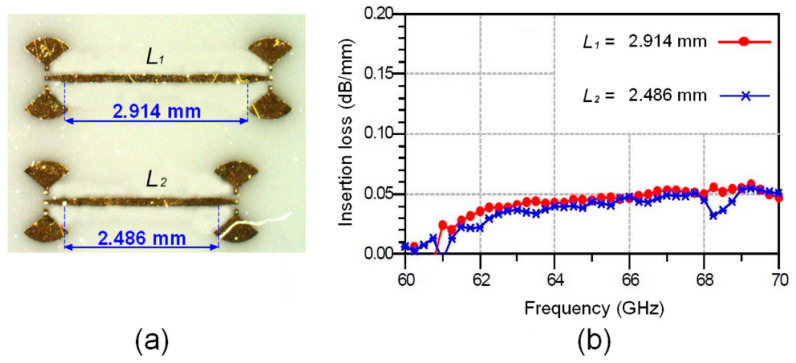
Microphotographs of both manufactured gold thin-film microstrip lines in (**a**) and the additional attenuation in the 60 GHz to 70 GHz frequency range in (**b**).

Figure 2a,b show, respectively, the microphotographs of both manufactured gold thin-film microstrip lines and the additional attenuation in the 60 GHz to 70 GHz frequency range. The considered frequency range starts from 60 GHz instead of 57 GHz (the starting frequency of the unlicensed 60 GHz frequency spectrum) due to the capability of measurement equipment (Keysight Technologies E8362B (VNA) connected with E-band extension modules, operating in the 60–90 GHz band).

The obtained results illustrated in Figure 2b show that the two curves are almost identical. The conductor losses of the 1 μm gold layer undergoing metallization do not exceed 0.05 dB/mm in the considered frequency range, from 60 GHz to 70 GHz. Given that the physical length of a quarter-wave line at the central frequency of 61 GHz is about 0.46 mm, we may conclude that the loss level is very low. These results demonstrate the success of the design and calibration, as well as the appropriate choice of substrate material.

## 4. Manufacturing Tolerance Analysis of the Thin-Film Resistive Layer

To experimentally assess the manufacturing tolerances of a thin-layer titanium oxide resistor (100 Ω per square), an MHMIC 50 Ω termination is implemented on an alumina ceramic substrate (ε_r_ = 9.5, *h* = 127 μm). This 50 Ω termination is a part of the circuit’s layout presented in Figure 1a, which includes some typical MHMICs such as 90-degree hybrid couplers, Wilkinson power dividers, and several identical TRL standard calibration kits.

**Figure 3:**
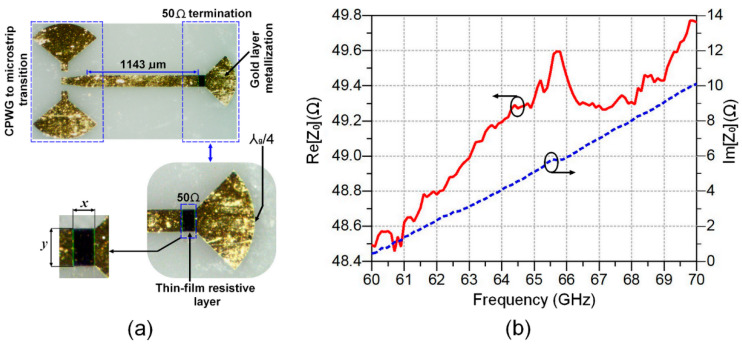
Microphotographs of the implemented MHMIC 50 Ω termination on an alumina substrate in (**a**) and the additional attenuation in the 60 GHz to 70 GHz frequency range in (**b**).

Figure 3a,b show an implemented MHMIC 50 Ω termination on an alumina ceramic substrate, and the results of on-wafer input impedance measurement (real and imaginary parts) versus frequency. As can be observed, the value of the real part of the input impedance ranges from 48.5 Ω to 49.8 Ω over the considered frequency range of 60 GHz to 70 GHz. However, the value of the imaginary part is very low around 61 GHz (the resonant frequency), and it increases with frequency to reach a value of 10 at 70 GHz. This increase in the value of the imaginary part is due to the presence of significant parasitic inductive effects at high millimeter-wave frequencies.

To estimate the maximum error during the implementation of a 100 Ω per square titanium oxide-based thin-layer resistor, we consider the fractional uncertainty of the area (*A*) in the 50 Ω rectangular resistor layer in Figure 3a. By considering that *x* and *y* are, respectively, the width and length of the titanium oxide rectangular covered area (*A*), the fractional uncertainty can then be expressed as follows [17,18]:

(2) $$\frac{∆A}{A_{0}}=\frac{∆x}{x}+\frac{∆y}{y}$$

where the area of the rectangle is described by the relation *A* = *x*∙*y*, and the initial area is represented by *A*_0_. Under those considerations, the final area is obtained by *A* = *A*_0_ ± ∆*A*.

In the case of a 100 Ω per square resistor, Equation (2) will be further simplified since *y* = *x* = *W*, where *W* is the microstrip line width. Then, Equation (2) becomes

(3) $$∆A=2A_{0}\cdot \frac{∆W}{W}$$

It should be noted that the values of *A*_0_ and *W* are, respectively, set at 0.0161 mm^2^ and 0.127 mm, taking account into the employed alumina substrate parameters. 

As can be seen from the Formula (3), the tolerance of the metallization process creates a variation ∆*W* in the microstrip line width, which, in turn, affects the tolerance level of a thin-layer resistor area ∆*A*. Therefore, by optimizing the accuracy of the gold metallization process, we may achieve a reduced error in the microstrip line width and, accordingly, enhance the implementation accuracy of a 100 Ω per square thin-layer resistor.

**Figure 4:**
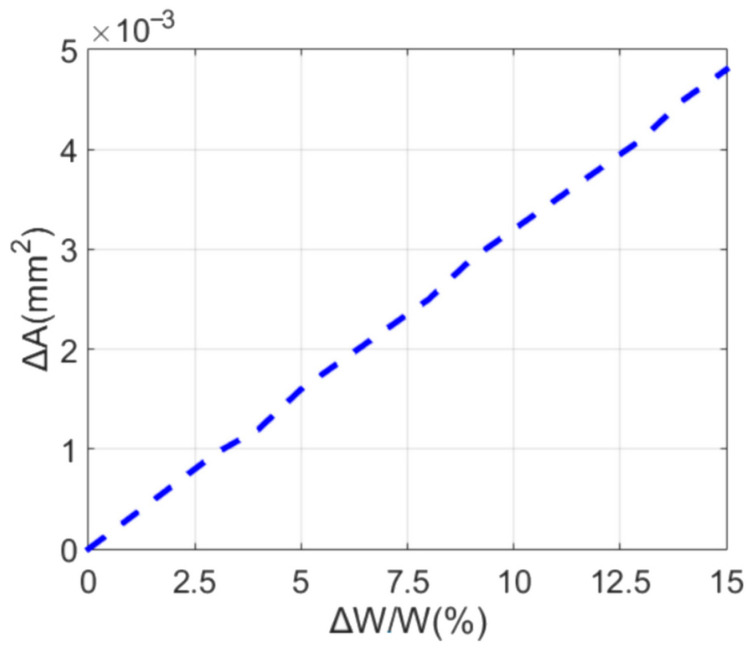
The thin-layer resistor area variation versus the relative deviation of microstrip line width.

The relationship in (3) allows for plotting curves of the thin-layer resistor area variation versus the relative deviation of the microstrip line width, as shown in Figure 4. The thin-layer resistor area variation increases linearly with the relative variation in microstrip line width. In general, the total thin-layer resistor area change does not exceed 0.0005 mm^2^ for a maximum relative variation in a microstrip line width of 15%.

## 5. Manufacturing Tolerance Analysis of a Quarter-Wavelength Radial Stub

Radial stubs are widely used in various microwave circuits such as filters, matching networks, biasing lines, and even grounding RF circuits. They provide a low impedance level at well-specified insertion points in a wide frequency band, contrary to the conventional straight stubs, which exhibit a reduced bandwidth and an increase in circuit size [19,20,21,22,23,24]. Now, we examine the accuracy improvement in the gold-metalized microstrip line that allows for manufacturing error reduction in the millimeter-wave microstrip quarter-wavelength radial stub. This radial stub technique enables performance optimization of the millimeter-wave grounding while avoiding via holes metallization [25,26,27].

**Figure 5:**
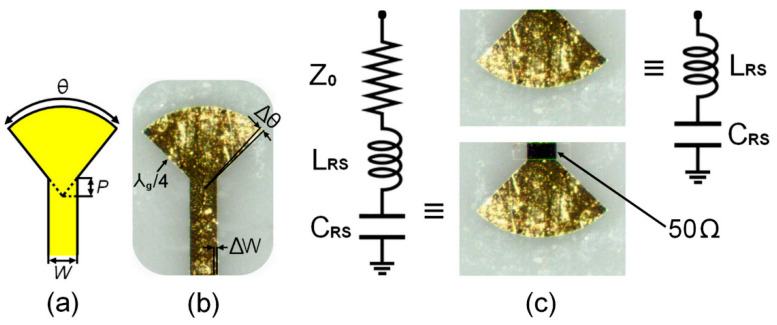
Millimeter-wave microstrip quarter-wavelength radial stub: (**a**) a quarter-wavelength radial stub with geometric parameters, (**b**) a microphotograph of the implemented prototype, and (**c**) the equivalent circuits with and without a 50 Ω load.

Figure 5a,b show the quarter-wavelength radial stub configuration including geometrical parameters and the microphotograph of the prototyped radial stub. The equivalent circuit of the quarter-wavelength radial stub with and without a 50 Ω termination is shown in Figure 5c. As can be observed, the radial stub with a 50 Ω load can be modelled by a series combination of a 50 Ω resistor, an inductor *L_rs_*, and a capacitor *C_rs_*, whereas the radial stub without a 50 Ω load is equivalent to an inductor *L_rs_* and capacitor *C_rs_* in series. 

According to Figure 5a, the geometrical parameters of the quarter-wavelength radial stub are as follows: *P* is the penetration depth, *W* is the microstrip width, and *θ* is the angle subtended by the stub, which is limited to a range of 10° ≤ *θ* ≤ 170°. For the proposed radial stub design, the values of those parameters have been set to *P* = 63.5 μm, *W* = 127 μm, and *θ* = 90°. 

The relative variation in the angle subtended by the stub and relative variation in the microstrip line width is governed by Equation (4) [28,29].

(4) $$\frac{∆\theta }{\theta }=\frac{1}{45}\cdot \arctan\left[W_{0}\cdot \left(\frac{1+\frac{∆W}{W}}{2\cdot P}\right)\right]-1$$

where *W*_0_ is the manufactured microstrip line width (*W*_0_ = 1.27 × 10^−4^ m).

**Figure 6:**
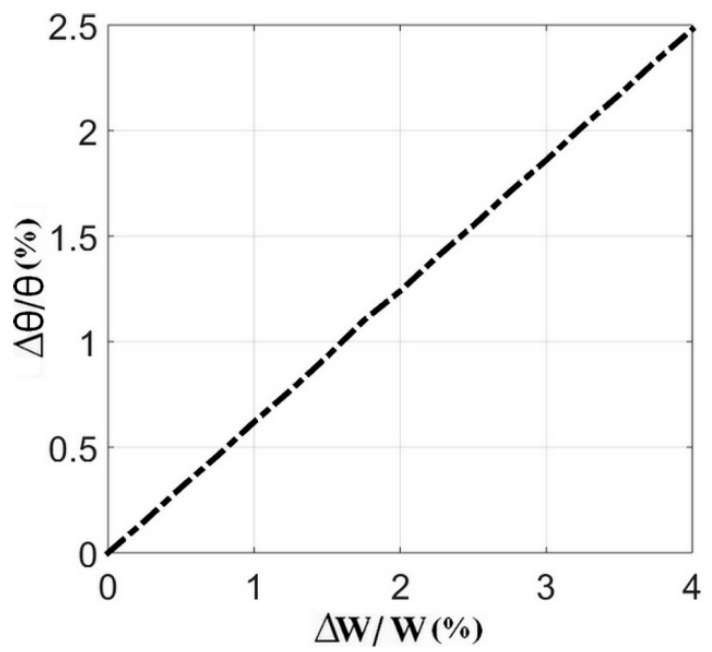
Relative variation in angle subtended by stub versus relative variation in microstrip line width for *ε_r_* = 9.5 and *h* = 127 μm.

It can be noted that the relative variation in the angle subtended by the stub could be optimized by minimizing the relative deviation in the microstrip line width. Using this equation, we can, therefore, plot the curves characterizing the relative variation in the angle as a function of the relative deviation of the microstrip line width, as shown in Figure 6 below. As a linear curve with a positive coefficient, it shows that for a maximum variation of 4% in the microstrip line width, the relative variation in the angle does not exceed 2.5%.

**Figure 7:**
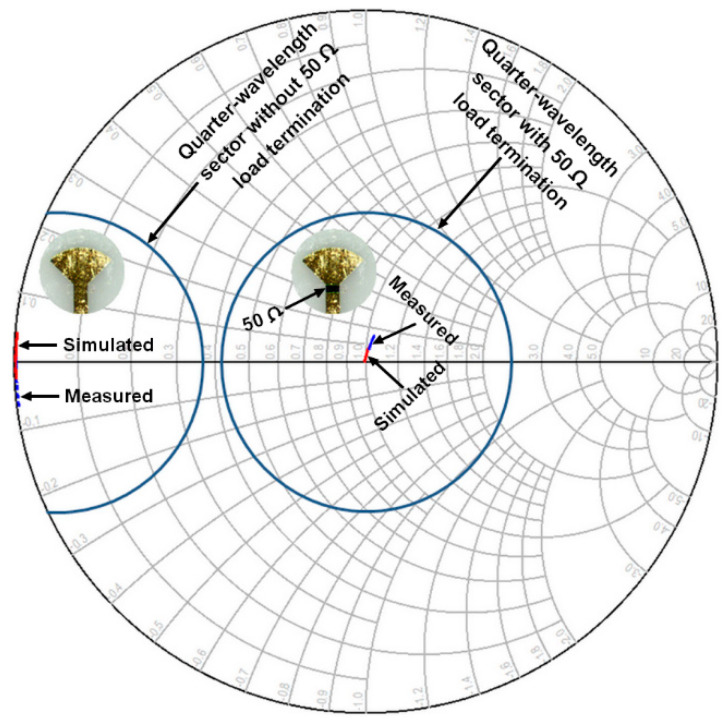
Measured and simulated impedance values on the Smith chart for the designed quarter−wavelength sector with and without the 50 Ω termination resistor for the 60 GHz to 65 GHz frequency range.

In order to verify the performances of short-circuit and 50 Ohm impedance matching for the designed quarter-wavelength sector, along with the 50 Ohm termination, a Smith Chart is employed to plot both the simulated and the measured results over the frequency range extending from 60 GHz to 65 GHz, as shown in Figure 7.

Mathematically, an ideal short circuit has typically zero impedance (zero resistance and reactance). Nonetheless, in reality, it is impossible to achieve that; the resistive part is usually so small and is approximated by zero Ohms. The measured and simulated short-circuit impedances at frequencies ranging from 60 GHz to 65 GHz are plotted on the left of the chart, at the intersection of the resistance and the reactance axes. However, by using the quarter-wave transformer, we can perform matching to a resistive load impedance of 50 Ohms, corresponding to the normalized load impedance of 1 at the center of Smith Chart. It should be noted that our design is optimized to operate around 61 GHz to enable experimental characterization with the available measurement equipment in our RF laboratory at INRS-EMT. As can be observed, a slight shift toward the inductive region occurred at the center. This parasitic inductive effect results from the microstrip transmission line acting as a series inductor. Overall, a reasonable agreement between measurements and simulations is achieved.

## 6. Manufacturing Tolerance Analysis of the Gold-Bonding Ribbon

At millimeter-wave frequencies, the interconnection between the chip devices and the RF circuits using the ribbon bonding technique has been identified as one of the key challenges due to the discontinuity introduced by the bond ribbon that can significantly affect the performance of the entire transceiver at the millimeter-wave frequency band. However, the ribbon bonding technique remains a very attractive solution in the electronic packaging domain since it is robust and cost-effective. Several studies on the transmission performance of ribbon bonding interconnection have been reported for microstrip and coplanar configurations. The reported studies specify that a bond ribbon could form a series inductor, contributing to drastically increasing losses, especially when the frequency or the bond ribbon length increases. For that reason, many efforts have focused on reducing the length of the bond ribbon, as well as reducing the chip-to-package gap to improve the interconnectivity performance at millimeter-wave frequencies [10,11,12].

**Figure 8:**
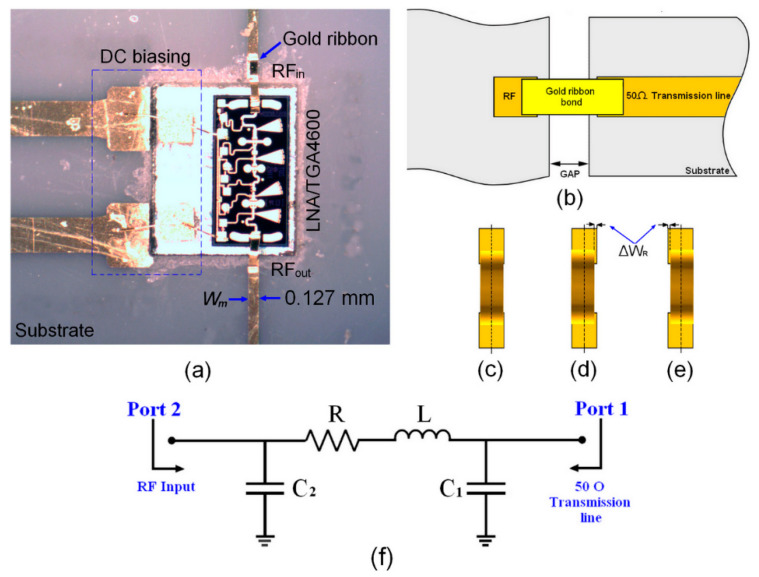
Microphotograph of the implemented TGA4600 low-noise amplifier (LNA) chip using ribbon bonding in (**a**). Typical ribbon bonding implementation in (**b**,**c**). Implementation imperfections in (**d**,**e**). The equivalent circuit model of ribbon bond in (**f**).

Figure 8a illustrates the ribbon bonding configuration of a highly integrated TGA4600 low-noise amplifier (LNA) chip with a 16-element antenna array in a 60 GHz MHMIC six-port RF front-end receiver. In this configuration, a 0.127 mm × 1 mm ribbon bond is employed to ensure an RF signal connection between the antenna array and the LNA RF_in_, as well as between the LNA RF_out_ and the six-port down converter input. 

Typical ribbon bonding implementation is shown in Figure 8b,c. However, the possible scenarios of imperfections in the ribbon bond implementation are shown in Figure 8d and Figure 8e, respectively.

The equivalent circuit model of the ribbon bond is shown in Figure 8f. It consists of resistance *R*, inductance *L*, and two distinct capacitors *C*_1_ and *C*_2_. The resistance R and the inductance *L* are related to the parameters of the bonding ribbon itself. However, the equivalent capacitances *C*_1_ and *C*_2_ at the ends of the bonding ribbon are not identical since the two ends of the bonding ribbon are connected to different materials. To accurately estimate the equivalent circuit model parameters, a circuit co-simulation is performed using the simulation tools of Keysight ADS software. The optimized parameters of this circuit model are selected as follows: *R* = 0.0061 Ω, *L* = 38.31 nH, *C*_1_ = 140.82 pF, and *C*_2_ = 124.61 pF.

**Figure 9:**
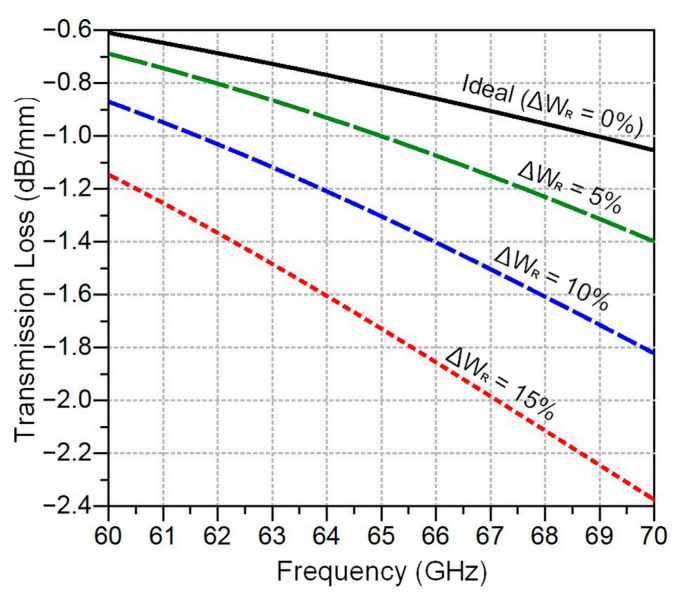
Transmission loss as a function of frequency for a 1 mm ribbon bond at different lateral deviation tolerances during ribbon bond implementation.

Figure 9 shows transmission loss as a function of frequency for a 1 mm ribbon bond length in different lateral deviation scenarios during ribbon bond implementation. It can be seen from the figure that in the case of a perfect implementation (∆W_R_ = 0%), the losses start from 0.6 dB/mm at 60 GHz to achieve a maximum of about 1 dB/mm around 70 GHz. However, a horizontal deviation of 5% in ribbon bond implementation generates extra losses of about 0.1 dB at 60 GHz and 0.35 dB at 70 GHz compared to the perfect scenario in Figure 8c. Overall, the transmission losses are more severe at high frequencies and significantly increase with the deviation tolerance to exhibit 1.15 dB/mm at 60 GHz and 2.4 dB/mm for a tolerance level of ∆W_R_ = 15%.

## 7. Conclusions

The implementation tolerances are critical parameters affecting the change in isolation, amplitude and phase balance for millimeter-wave MHMIC circuits. In this paper, a tolerance analysis is performed to investigate the sensitivity of the MHMIC fabrication process to manufacturing tolerances over the unlicensed 60 GHz Industrial–Scientific–Medical (ISM) band. The results have demonstrated that the MHMIC fabrication technique enables reasonable tolerances at millimeter-waves for passive MHIMICs. However, further optimization of these tolerances is feasible by optimizing the implementation accuracy of thin-film gold microstrip transmission lines. This study provides key inputs for the improvement of the MHMIC manufacturing process and enables designers to consider the critical parameters affecting the performances of the designed circuits at millimeter-wave frequencies.

## Data Availability

Data generated during the study are contained within the article.
